# simpleNomo: A Python Package of Making Nomograms for Visualizable Calculation of Logistic Regression Models

**DOI:** 10.34133/hds.0023

**Published:** 2023-06-07

**Authors:** Haoyang Hong, Shenda Hong

**Affiliations:** ^1^ National Institute of Health Data Science, Peking University, Beijing, China.; ^2^ School of Data Science, Chinese University of Hong Kong, Shenzhen, China.

## Abstract

**Background:**

Logistic regression models are widely used in clinical prediction, but their application in resource-poor settings or areas without internet access can be challenging. Nomograms can serve as a useful visualization tool to speed up the calculation procedure, but existing nomogram generators often require the input of raw data, inhibiting the transformation of established logistic regression models that only provide coefficients. Developing a tool that can generate nomograms directly from logistic regression coefficients would greatly increase usability and facilitate the translation of research findings into patient care.

**Methods:**

We designed and developed simpleNomo, an open-source Python toolbox that enables the construction of nomograms for logistic regression models. Uniquely, simpleNomo allows for the creation of nomograms using only the coefficients of the model. Further, we also devoloped an online website for nomogram generation.

**Results:**

simpleNomo properly maintains the predictive ability of the original logistic regression model and easy to follow. simpleNomo is compatible with Python 3 and can be installed through Python Package Index (PyPI) or https://github.com/Hhy096/nomogram

**Conclusion:**

This paper presents simpleNomo, an open-source Python toolbox for generating nomograms for logistic regression models. It facilitates the process of transferring established logistic regression models to nomograms and can further convert more existing works into practical use.

## Introduction

Logistic regression is a widely used machine learning model for predicting binary outcomes, including in medical diagnosis [1,2]. In some cases, it has been shown to perform as well as artificial neural networks [2]. However, the logistic regression model involves complex calculations, which can be computationally demanding and inconvenient to use in clinical situations that require rapid diagnosis and lack computational resources, especially in underdeveloped regions. As a result, there is a need for tools that can help simplify the process of using logistic regression models in clinical practice, such as nomograms [3], which can provide a visual representation of the model’s predictions and facilitate rapid interpretation by clinicians.

**Fig. 1.**
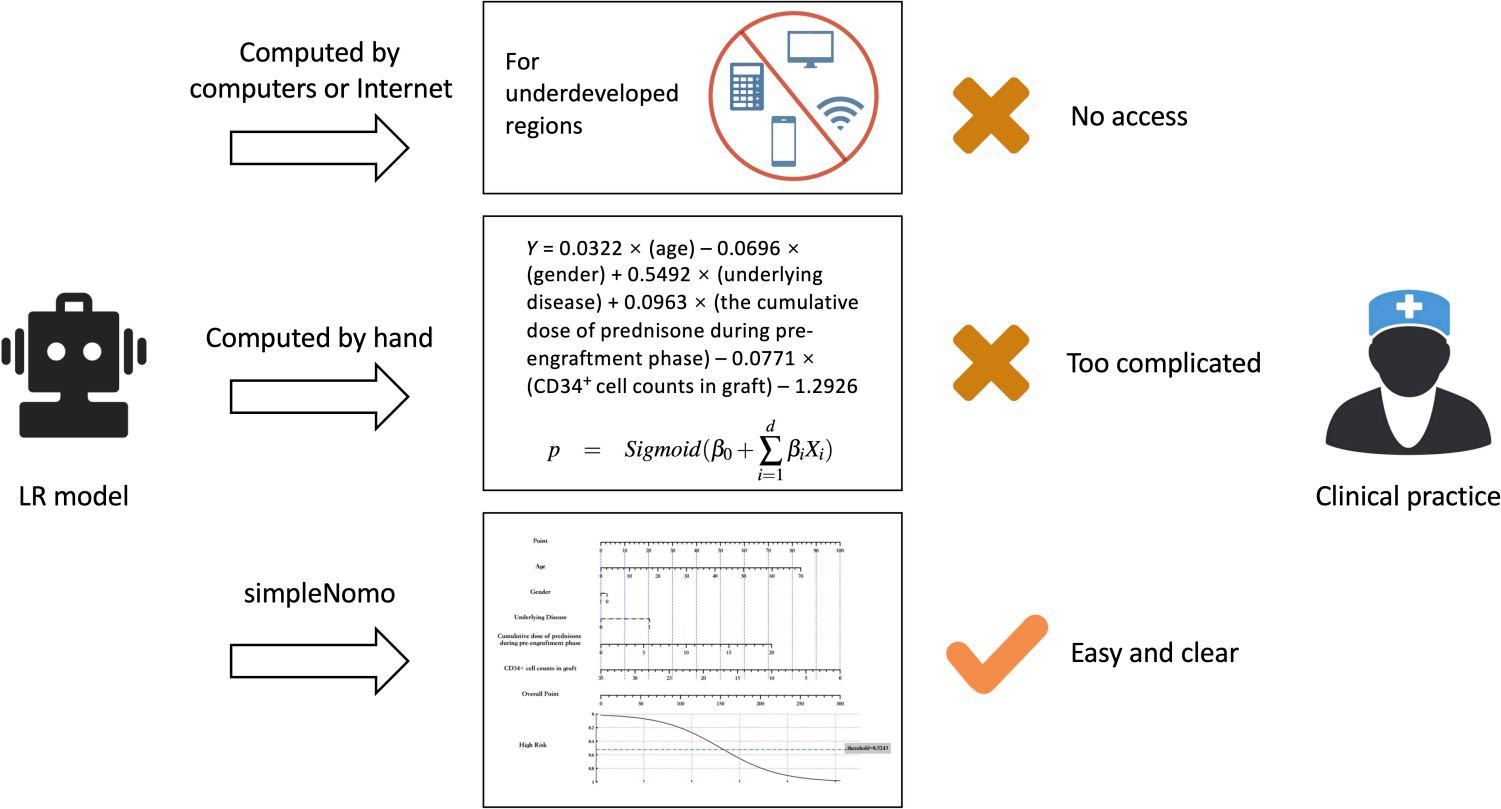
Situations where nomograms come in convenience.

Nomograms are graphical calculators that have been used to visualize logistic regression models since their invention by Philbert Maurice d’Ocagne in 1880 [4,5]. They consist of several arranged lines that can be used to perform fast graphical calculations of complex formulas. Although nomograms have become less popular since the advent of electronic calculators and computers, they remain a valuable tool in situations where computational resources are limited or unavailable (Fig. 1). One of the main advantages of nomograms is that they can be carried anywhere and reused without the need for recalculating the results [6]. A considerable body of literature has demonstrated the development of nomograms based on logistic regression models in the field of clinical research [7–10], depicting the prevalence of nomograms as a tool in clinical practice. There are several software tools available for generating nomograms, including SAS [11], Stata [12], as well as the rms [13] and hdnom [14] packages in the R programming language.

However, these software tools have limitations in their input requirements. SAS lacks an integrated toolbox for generating nomograms from logistic regression models, while hdnom and rms only accept inputs from models trained within the R programming language. This presents a challenge for researchers to transfer developed logistic regression models that did not publish the training data to nomograms. As a result, many potentially valuable models may remain unused in clinical practice. To address this challenge, we developed simpleNomo, an open-source Python toolbox that enables the direct construction of nomograms from logistic regression coefficients. By providing a user-friendly and efficient means of generating nomograms, simpleNomo has the potential to facilitate the translation of research findings into clinical practice and improve patient care.

Unlike other tools, simpleNomo accepts only the coefficients and range of predictors in a logistic regression model as input, making it easier to obtain the necessary information for nomogram construction. This feature allows researchers to easily transfer existing logistic regression models into nomograms that can be utilized in a wider range of clinical applications. Our work presents a valuable contribution to the field of clinical prediction modeling application and has the potential to improve patient care by facilitating the translation of research findings into practical use.

## Design and Development

In this section, we demonstrate how to construct a nomogram directly from logistic regression model coefficients and predictor ranges. The nomogram assigns a point value to each predictor based on its value, and the sum of these points is used to calculate the predicted probability of the outcome.

### Problem formulation

Assume we want to transfer a logistic regression model consisting *d* predictors with Eq. 1, where *β_i_* represents the *i*-th coefficient, *X_i_* represents the *i*-th predictor, and *p* represents the probability of getting positive result (*y* = 1). The transformation procedure consists of 2 steps, point assignment and probability assignment. The notations are summarized in Table 1.

$$p=\mathrm{Sigmoid}\left(\beta_{0}+\sum_{i1}^{d}\beta_{i}X_{i}\right)$$ (1)

$$y=\left\{\begin{array}{l} 1\;p>\theta \\ 0\;p<\theta \end{array}\right.$$ (2)

**Table 1.** Notation and corresponding explanation.

Notation	Meaning
*d*	Number of predictors
*X_i_*	*i*-th predictor
*y*	Label; e.g., high/low risk (usually represented by 1/0)
*p*	Probability for high risk (i.e., *y* = 1)
*β*_0_	Intercept for the linear model
*β_i_*	Coefficient for *i*-th predictor
*θ*	Threshold for the regression model (i.e. *y* = 1 if *p* > *θ*)
$P_{i}^{\max}$	The maximum point assigned to the *i*-th predictor
*P^max^*	$\underset{i}{\arg}\max\;P_{i}^{\max}$ (usually 100)
*P*_*i*_*v*_	The point assigned to value *v* of predictor *X_i_*
*sigmoid* (·)	Sigmoid function i.e $\text{sigmoid}\;\left(x\right)=\frac{1}{1+\exp\left(-x\right)}$
*max_i_*	Maximum value for *i*−th predictor
*min_i_*	Minimum value for *i*−th predictor
*range_i_*	*max_i_* − *min_i_*

### Point assignment

Firstly, we initialize the point assignment through choosing an initializing predictor. For each predictor, compute its absolute maximum beta value according to Eq. 3. We choose the predictor with the largest absolute maximum beta value to be the initializing predictor (denoted as *X_i_*). If *β_i_* ≥ 0, assign its maximum value to *P^max^* and its minimum value to 0. If *β_i_* < 0, assign its maximum value to 0 and its minimum value to *P^max^*. As for the other values *v* of this predictor, assign the point according to Eq. 4.

$$\text{Absolute maximum beta value}=\left|\beta_{i}*{\text{range}}_{i}\right|$$ (3)

$$\text{Assigned point}=\left\{\begin{array}{c} \frac{v-\min_{i}}{\max_{i}{-\min}_{i}}\;P_{i}^{\max}\;\beta_{i}\geq 0\\ \frac{\max_{i}-v}{\max_{i}-\min_{i}}\;P_{i}^{\max}\;\beta_{i}<0 \end{array}\right.$$ (4)

After the initialization, for each predictor *X_j_* (*j* ≠ *i*), compute the maximum assigned point according to Eq. 5. If *β_j_* ≥ 0, assign its maximum value to $P_{j}^{\max}$ and its minimum value to 0. If *β_j_* < 0, assign its maximum value to 0 and its minimum value to $P_{j}^{\max}$. As for the other values *v* of this predictor, assign the point according to Eq. 4. The algorithm can be summarized in Algorithm 1.

$$P_{j}^{\max}=P^{\max}\left|\frac{\beta_{j}*{\text{range}}_{j}\;}{\beta_{i}*{\text{range}}_{i}}\right|$$ (5)

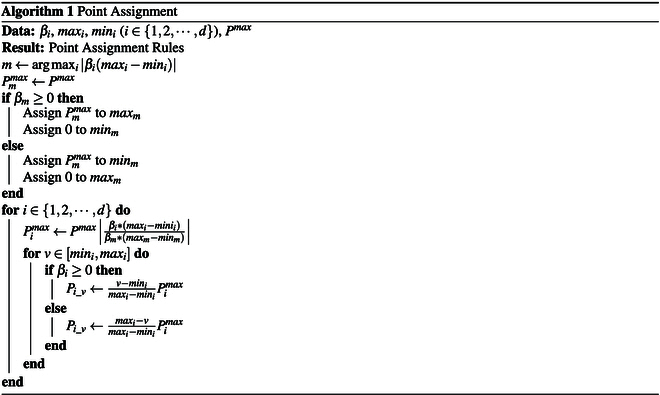


### Probability assignment

After the point assignment procedures, we get the mapping from predictors’ values to a specific point. In the probability assignment part, we need to assign the sum of all predictors’ points to a probability. Firstly, we compute the range of the sigmoid function input according to Eqs. 6 and 7. According to the point assignment algorithm, the point assigned for each predictor is from 0 to $P_{i}^{\max}$. Therefore, the minimum overall point (summation of all predictors’ points) is 0 and the maximum overall point P_total_max is computed as Eq. 8. Therefore, for each overall point P_total ranging in 0 and P_total_max, it can be mapping to a value *v* that is the input of the sigmoid function through Eq. 9. Therefore, the assigned probability can be computed by *sigmoid(v)*. The algorithm can be summarized in Algorithm 2.


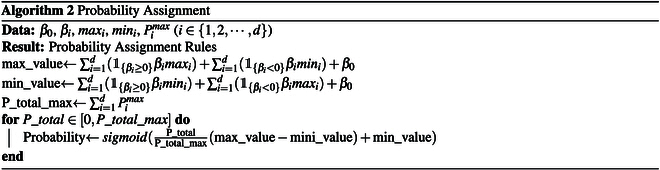


**Fig. 2.**
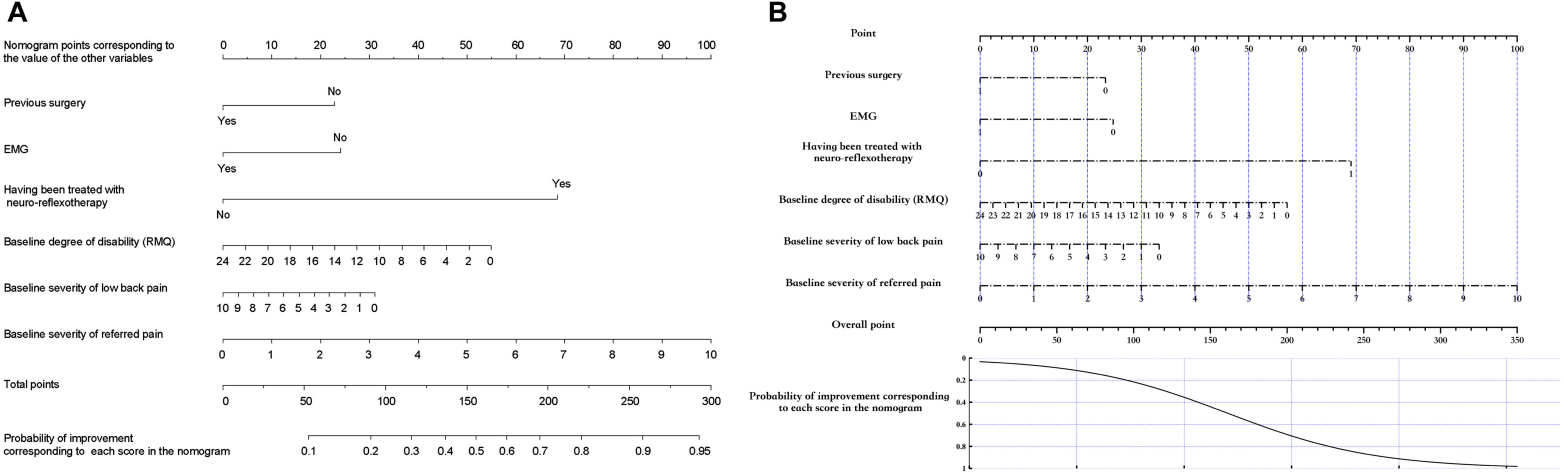
Nomogram styles comparisons between (A) existing nomogram methods [17] and (B) proposed nomogram generating methods simpleNomo.

We proposed a modification to current nomograms by addressing the issue of uneven-scaled axes in probability assignment. Specifically, we suggest using a sigmoid curve to map the sum of all predictors’ points to the probability instead of the existing method that employs an uneven-scaled axis (Fig. 2A). This approach results in a more accurate assignment of probability by ensuring that the y-axis, which represents probability, is uniformly distributed (Fig. 2B); the nomogram generation procedure is shown in B.1. Our proposed modification thus offers an improvement over current nomograms in terms of accuracy of probability assignment.

$$\begin{array}{c} \text{min\_value}=\sum_{i=1}^{d}\left(1_{\left\{\mathrm{Bi}\geq 0\right\}}\beta_{i}\min_{i}\right)+\\ \;\sum_{i=1}^{d}\left(1\left\{\beta_{i}<0\right\}\beta_{i}\max_{i}\right)+\beta_{0} \end{array}$$ (6)

$$\begin{array}{c} \text{max\_value}=\sum_{i=1}^{d}\left(1_{\left\{\beta_{i}\geq 0\right\}}\beta_{i}\max_{i}\right)+\\ \;\sum_{i=1}^{d}\left(1_{\left\{\beta_{i}<0\right\}}\beta_{i}\min_{i}\right)+\beta_{0} \end{array}$$ (7)

$$\text{P\_Total\_max}=\sum_{i=1}^{d}P_{i}^{\max}$$ (8)

$$\frac{\text{P\_Total}-0}{\text{P\_total\_max}-0}=\frac{v-\text{min\_value}}{\text{max\_value}-\text{min\_value}}$$ (9)

### Axis design

**Fig. 3.**
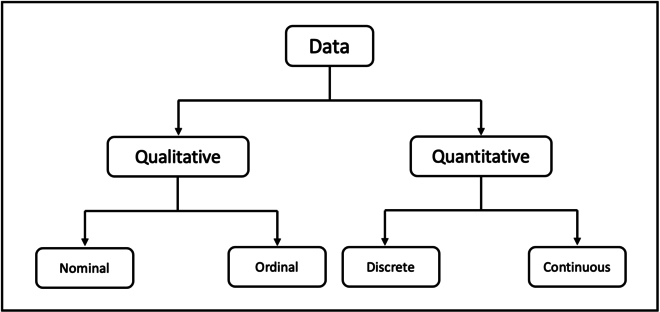
Data type.

There are 2 types of data included in linear model construction: qualitative and quantitative data, which are further classified into 4 types: nominal, ordinal, discrete, and continuous (Fig. 3). During the nomogram design process, we carefully consider the data types to ensure that the resulting nomogram effectively displays the relevant information.

1. Nominal data, which is used to label variables without order or quantitative values, is represented in simpleNomo using dashed lines to indicate that the values are discrete and limited. In linear models, nominal data is typically transformed into several binarized variables using one-hot encoding to facilitate analysis. However, this can lead to a large number of axes and redundancies in the resulting nomogram. To address this issue, simpleNomo integrates binarized variables belonging to the same nominal variable into a single dashed line, as they are mutually exclusive. This approach simplifies the nomogram design and improves its clarity, making it easier for clinicians to interpret and utilize the results.
2. In simpleNomo, the axis for continuous data is represented by a solid line to indicate that the values can vary continuously between their minimum and maximum values.

### Online tools development

**Fig. 4.**
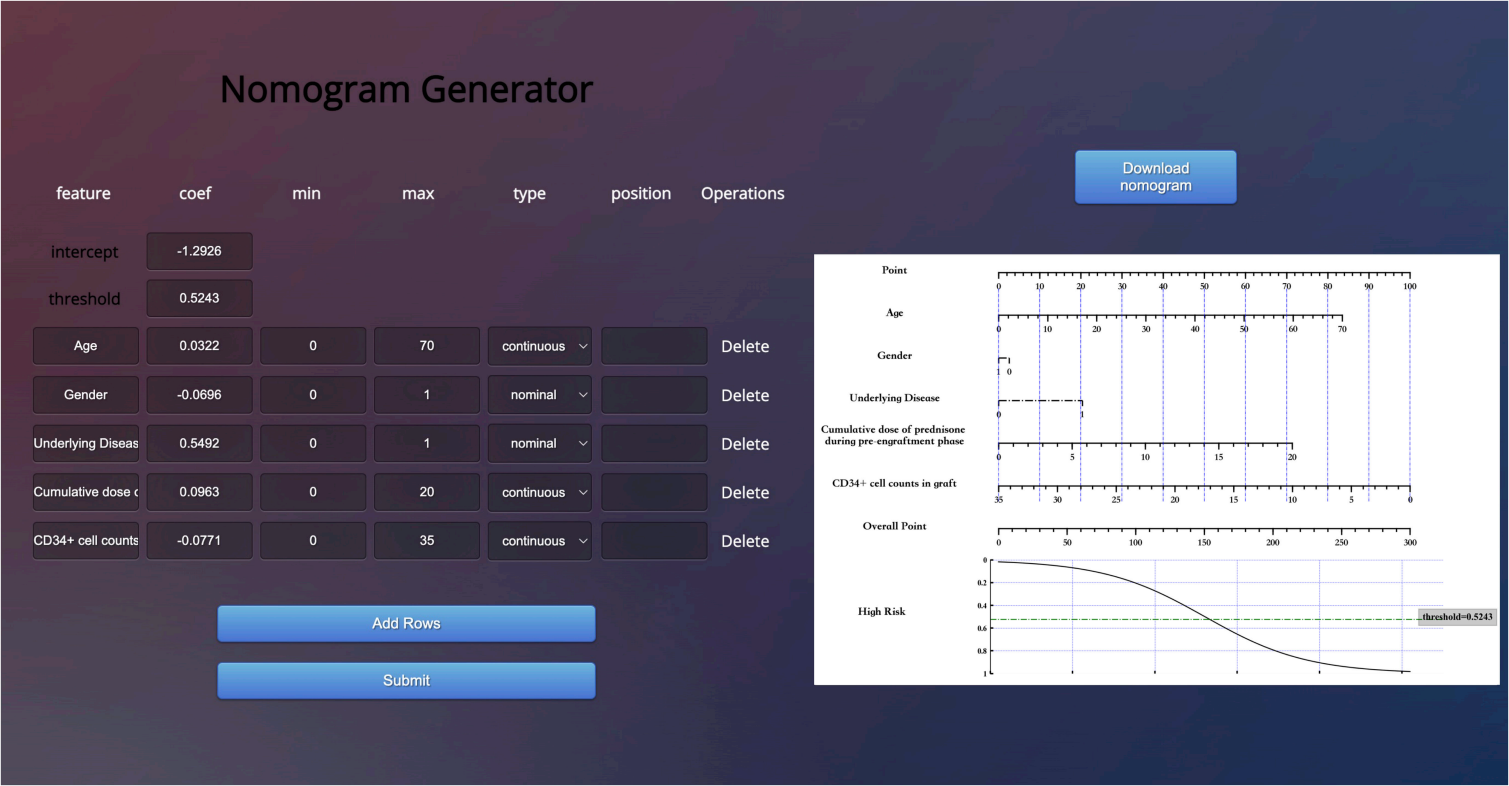
Website of nomogram generator.

In addition, we have developed an online tool (Fig. 4) to simplify the process of developing nomograms for clinicians. Users can easily fill in the required information in a similar manner as filling in an Excel template, and then simply click the “Submit” button to generate their own nomograms. We developed the web server with Tornado based on the simpleNomo code.

## Validation and Evaluation

We validated and evaluated the performance of nomograms for clinical use from both theoretical and quantitative perspectives.

### Theoretical analysis

**Fig. 5.**
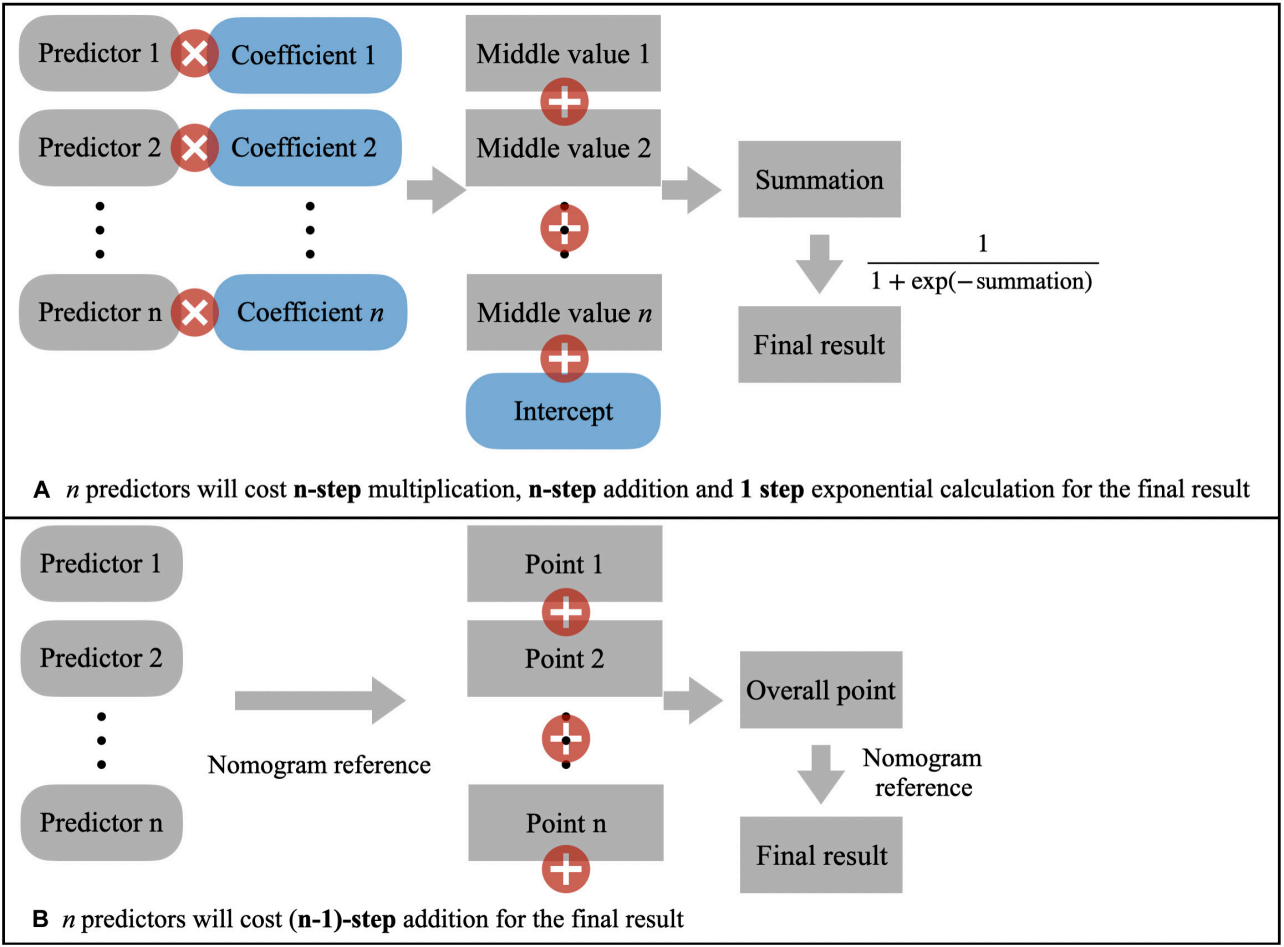
Calculation flow for (A) original logistic regression model formula. (B) Nomogram for the same logistic regression model.

Figure 5 demonstrates how nomograms simplify the computation process for logistic regression models. Given a well-trained logistic regression model with *n* predictors, several steps are required to calculate the predicted outcome by hand for a given instance. This includes *n* steps to calculate the multiplication of the coefficients and instance values for each predictor, followed by *n* steps to sum up the resulting middle values with the model intercept, and finally calculating the predicted outcome using an exponential function, $\frac{1}{1+\text{exp}\;\left(-\text{summation}\right)}$. This process involves *n* multiplications, *n* additions, and 1 exponential calculation. In contrast, a nomogram for the same logistic regression model with *n* predictors and given instance values first refers to the *n* instance values to assign corresponding points on the nomogram. The values of these points are then summed up to obtain an overall point, which is used to determine the predicted outcome. Thus, nomograms replace the complicated multiplication and exponent operations in the computation process with graphical reference, greatly simplifying the calculation process and improving the efficiency of logistic regression model prediction.

### Quantitative analysis

In our evaluation of the nomogram’s performance, we follow the quantitative evaluation criteria suggested by Balachandran et al. [15]. This research proposed that the evaluation of a nomogram’s performance should consist of 3 components: discrimination, calibration, and clinical usefulness. Discrimination refers to the ability of the nomogram to distinguish between patients who experience an event and those who do not. Calibration assesses how closely the nomogram-user estimated risk aligns with the risk computed by the logistic regression model, which can be depicted using a calibration plot. The final component in the evaluation of nomogram performance is clinical usefulness, which involves validating whether the use of the nomogram in decision-making improves patient outcomes. By considering these 3 components, we aim to provide a comprehensive evaluation of the nomogram’s performance and its potential impact on clinical practice.

In our evaluation of the nomogram’s performance, we focused on the discrimination and calibration criteria described earlier. To assess these criteria, we constructed a questionnaire that contained 5 different nomograms, each representing a patient case with 2 questions to fill: the estimated risk using the nomogram and whether the patient is at high risk or not. The questionnaire contained a total of 10 questions, which we distributed to 31 volunteers. Of the 31 volunteers, 30 completed the entire questionnaire, while 1 volunteer answered 8 out of 10 questions.

**Fig. 6.**
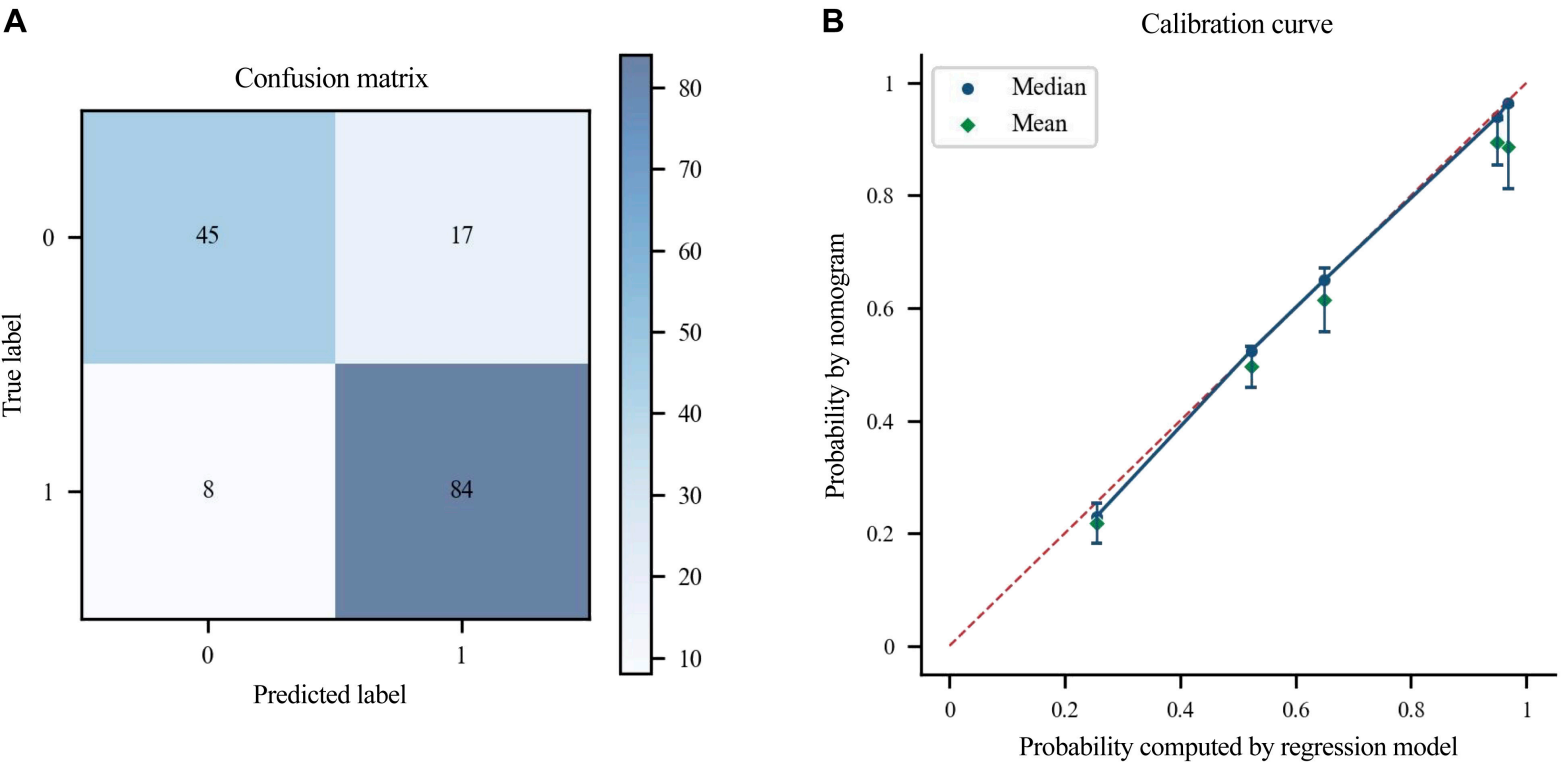
(A) Confusion matrix of nomograms estimating results. (B) Calibration curve of nomograms estimating probability. Error bars = 95% confidence intervals.

Thirty-one volunteers generated 154 answers for estimated probability and 154 answers for the high- or low-risk judgment. Among the answers for high or low risk judgment, 129 out of 154 were consistent with the result predicted by the original logistic regression model, which achieves an accuracy of 0.8377. The falsely predicted cases are all centered on the case with a predicted probability only 0.02 less than the threshold. To further demonstrate the sound performance of the nomograms, we evaluated their calibration performance using a calibration curve for the estimated probability, as shown in Fig. 6. The curve showed relatively small confidence intervals for the estimated error, indicating good calibration performance for the generated nomograms. These results provided strong evidence for the effectiveness and potential clinical usefulness of the nomograms in predicting patient outcomes.

## Comparison

The current tools for developing nomograms based on logistic regression models either lack an integrated toolbox, such as SAS, or only accept input from models trained within a specific programming language, such as hdnom and rms. As a result, converting existing logistic regression models that lack published training data or nomograms is challenging. Consequently, many models with good performance may not have meaningful clinical applications, and their development may stagnate. A summary comparison of the available tools is presented in Table 2.

**Table 2.** Difference between different nomograms generation tools.

Tools	SAS [11]	Stata [12]	rms [13]	hdnom [14]	simpleNomo
Programming languages	SAS	Stata	R language	R language	Python
Integrated packages or not		√	√	√	√
User interface		√			√
Probability matching curve					√
Based only on model coefficients	√				√
Self-designed point range	√	√			√

## Using Instruction

**Fig. 7.**
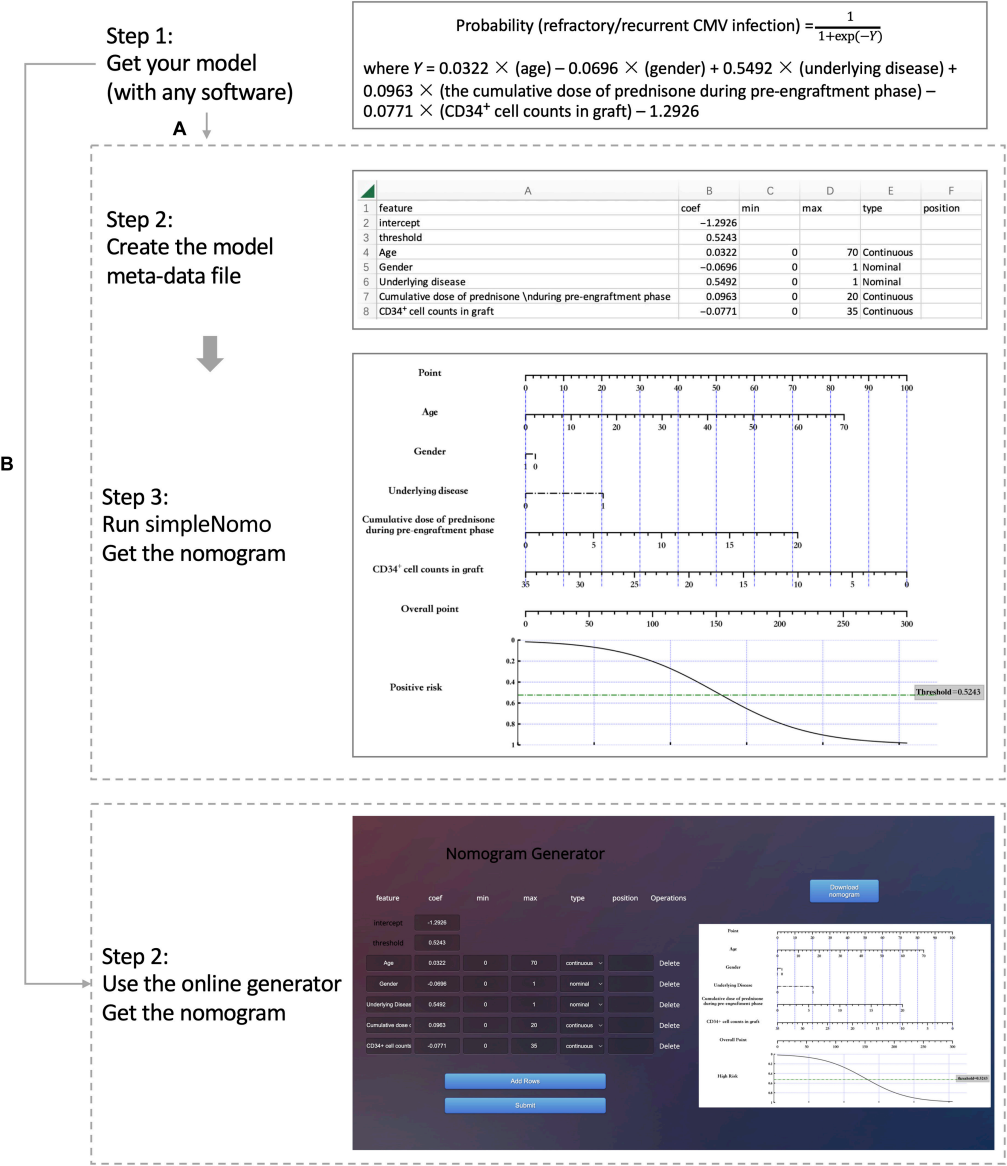
Workflow for constructing nomograms based on established logistic regression model. (A) Use simpleNomo package. (B) Use online generator.

We present 2 methods for generating nomograms based on established logistic regression models (see Fig. 7). The first approach involves using simpleNomo package, while the second approach entails using the online generator. simpleNomo is compatible with Python3; it relies on numpy, pandas, and matplotlib.

### Step 1: Get your model with any software

An example can be illustrated as follows. In [16], Hong et al. constructed a logistic regression model to predict refractory/recurrent cytomegalovirus (CMV) infection after haploidentical donor (HID) hematopoietic stem cell transplantation (HSCT). The model is presented as

$$\begin{array}{c} \text{Probability}\;\left(\text{refractory}/\text{recurrent}\;\mathrm{CMV}\;\text{infection}\right)=\\ \;\frac{1}{1+\exp\;\left(-Y\right)} \end{array}$$

where *Y* = 0.0322 × (age) − 0.0696 × (gender) + 0.5492 × (underlying disease) + 0.0963 × (the cumulative dose of prednisone during pre-engraftment phase) − 0.0771 × (CD34^+^ cell counts in graft) − 1.2926. The threshold of probability was 0.5243, which separates patients into high- and low-risk groups.

### Option 1: Use simpleNomo package

#### Step 2: Create the model meta-data file

**Fig. 8.**
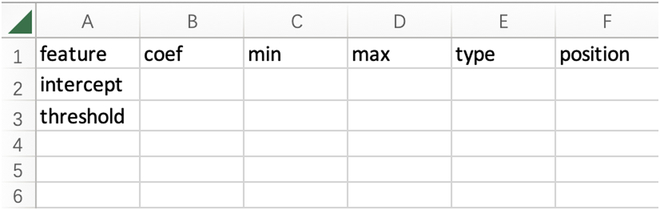
Template of meta-data file.

According to the presented model and variable description in [16], the meta-data file for generating nomogram can be filled as the chart in Fig. 7. The template for filling is shown in Fig. 8.

To facilitate the nomogram construction, it only needs to take input from an organized excel. The template for the excel chart can be downloaded from https://github.com/Hhy096/nomogram. It consists of 6 columns which are feature, family coef, min, max, type, and position.

1. feature: Names for variables. The first 2 elements of feature are intercept for logistic regression and threshold for distinguishing 2 cases. If there is no threshold, the threshold can be left as blank.
2. coef: Coefficients for variables.
3. min: Minimum possible value for variables.
4. max: Maximum possible value for variables
5. type: Data type for variables, it takes value from continuous, discrete, nominal and ordinal.
6. position: It takes value from "up" and "down". "up" ("down") represents that the tick labels for corresponding variable is above (below) the line.

#### Step 3: Run simpleNomo and get the nomogram

The code for generating nomogram and results are shown in code and Fig. 7 respectively.


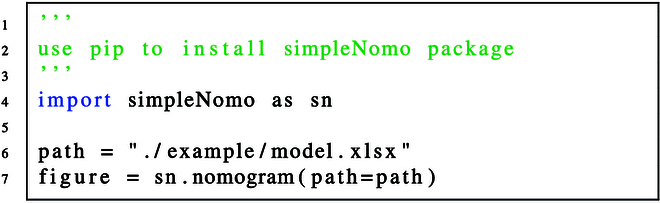


### Option 2: Use online generator

The online generator can be found on https://github.com/Hhy096/nomogram. To use the online generator, users can fill in the required information in the same manner as generating the meta-data file, as described in the previous section. After completion of the necessary fields, users can click the ’Submit’ button to generate the corresponding nomogram.

## Application Examples

**Fig. 9.**
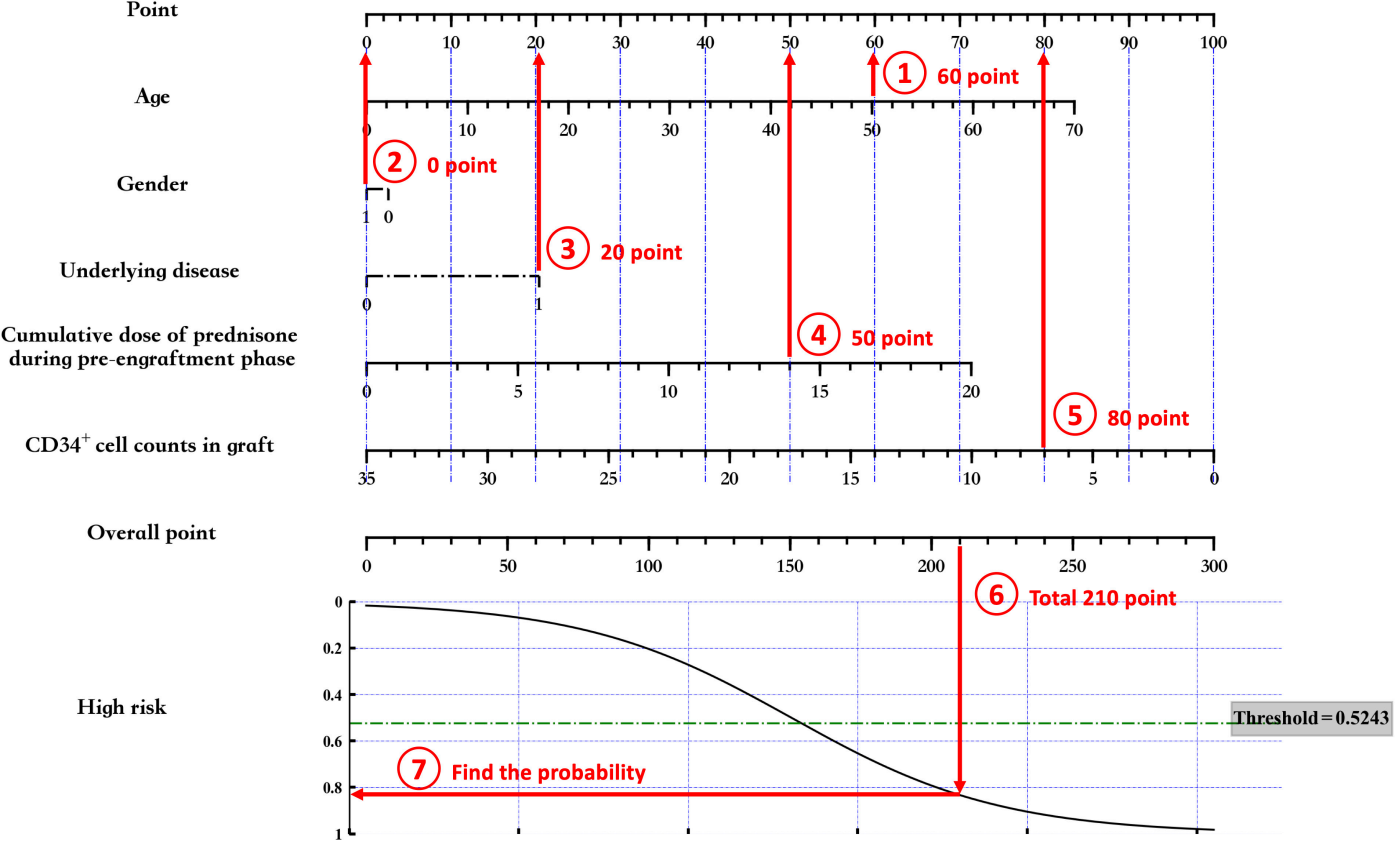
Example for utilizing nomogram in practice.

Once a nomogram has been generated, the next step is to apply it in practical use. As an example, consider the nomogram shown in Fig. 7, which was constructed using the simpleNomo framework. To use this nomogram to predict the risk of CMV infection after HID HSCT for a patient with the following values: Age = 50, Gender = 1, Underlying Disease = 1, Cumulative dose of prednisone during pre-engraftment phase = 14, and CD34^+^ cell counts in graft = 7, we first assign each value to its corresponding point on the nomogram, as shown in Fig. 9. The resulting point values are 60, 0, 20, 50, and 80, respectively. Summing these values yields an overall point value of 210, which can be used to determine the risk of CMV infection through the curve provided in the nomogram. Based on this calculation, the predicted risk of CMV infection after HID HSCT for this patient is 0.82. This example demonstrates the practical application of nomogram in predicting clinical outcomes.

## Data Availability

simpleNomo is compatible with Python 3 and can be installed through Python Package Index (PyPI) or https://github.com/Hhy096/nomogram.

## Appendices

**A. Change the style of nomograms**

**Fig. 10.**
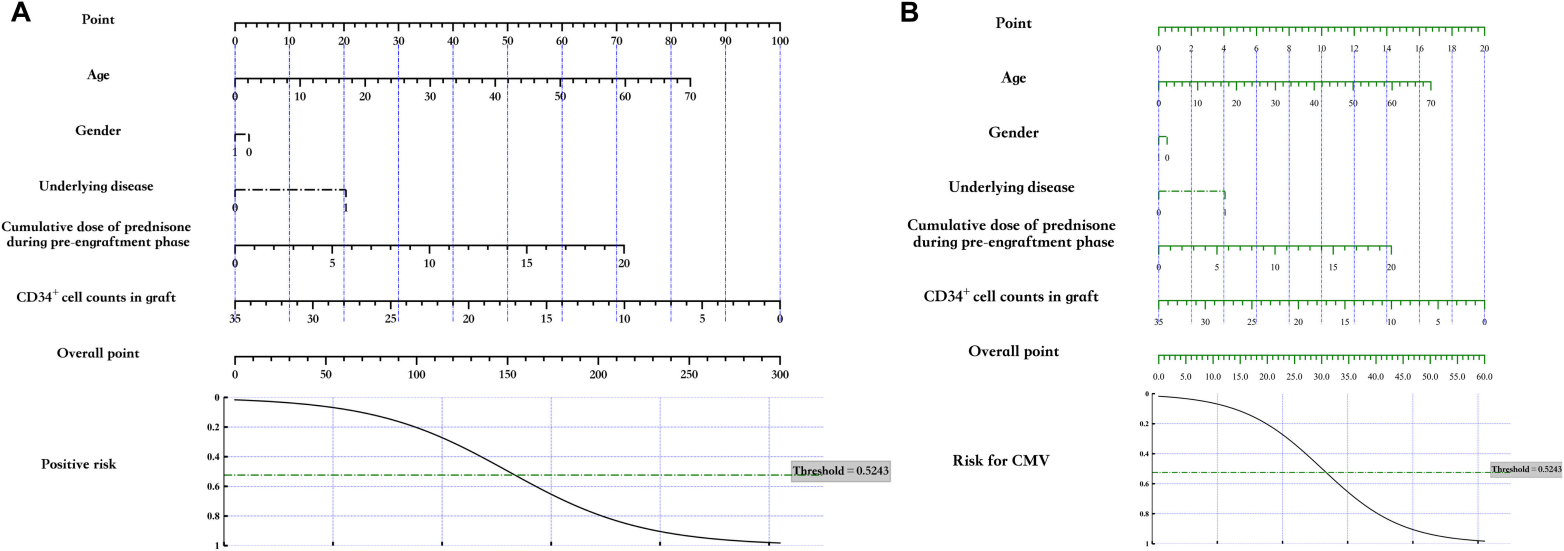
Change the style of the nomograms representing the same logistic regression model shown in Step 1: Get your model with any software. (A) The nomogram without any manipulation. (B) The nomogram generated through code A.

The users can simply change the nomogram style by adding parameters to the function in simpleNomo. For example, if the users want to change the nomogram shown in Fig. 7 Step 3, they can use the same excel file as in Fig. 7 Step 2, and the following parameters can be added and the result is shown in Fig. 10.


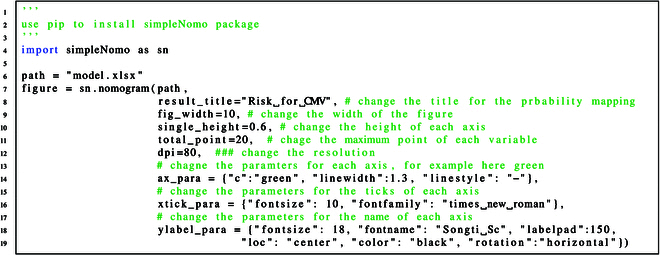


**B. Nomogram development for established logistic regression model**

In this study, we provided examples of constructing nomograms based on several published logistic regression models that do not currently include nomograms. Specifically, we selected the evolution of low back pain patients prediction model [17], the Epstein-Barr virus reactivation (EBV) prediction model [18], the refractory/recurrent CMV infection prediction model [16], the severe acute graft-versus-host disease (aGVHD) prediction model [19], and the risk of mortality in pediatric intensive care unit (ICU) prediction model [20]. To construct the nomograms, we followed a sequential process of retrieving logistic regression model information from the published papers, constructing the meta-data file, and developing the nomogram using either the simpleNomo software or the online generator.

**B.1 Nomogram generation for low back pain prediction models**

Our work offers a valuable contribution to clinical practice by enabling the construction of nomograms based on published logistic models. The way for constructing nomogram based on the coefficients and predictors’ range is shown as follows.

**Fig. 11.**
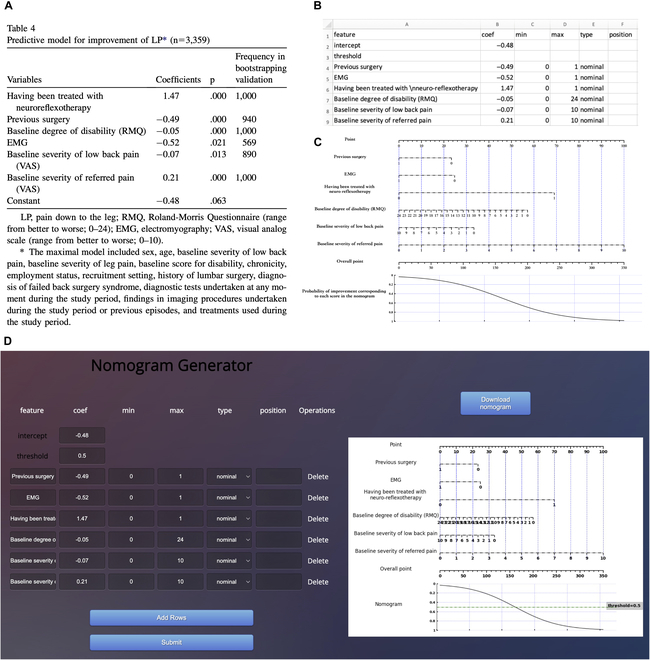
Develop nomograms from established logistic regression model. (A) The original presentation of logistic regression model information [17]. (B) The excel containing the logistic regression model information for generating nomogram. (C) The nomogram generated by simpleNomo. (D) Generate the nomogram with an online generator.

Kovacs et al. [17] presented the model coefficients and predictors’ range as in Fig. 11A. Accordingly, we can generate the meta-data file containing the necessary information, as depicted in Fig. 11B, and save it as model.xlsx. Then run the code in B.1, it will generate the nomogram shown in Fig. 11C. If the user chooses to use online generator, Fig. 11D shows how to construct the nomogram.


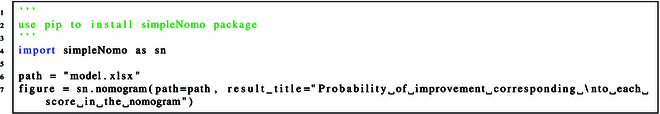


**B.2 Nomogram for EBV prediction**

**Fig. 12.**
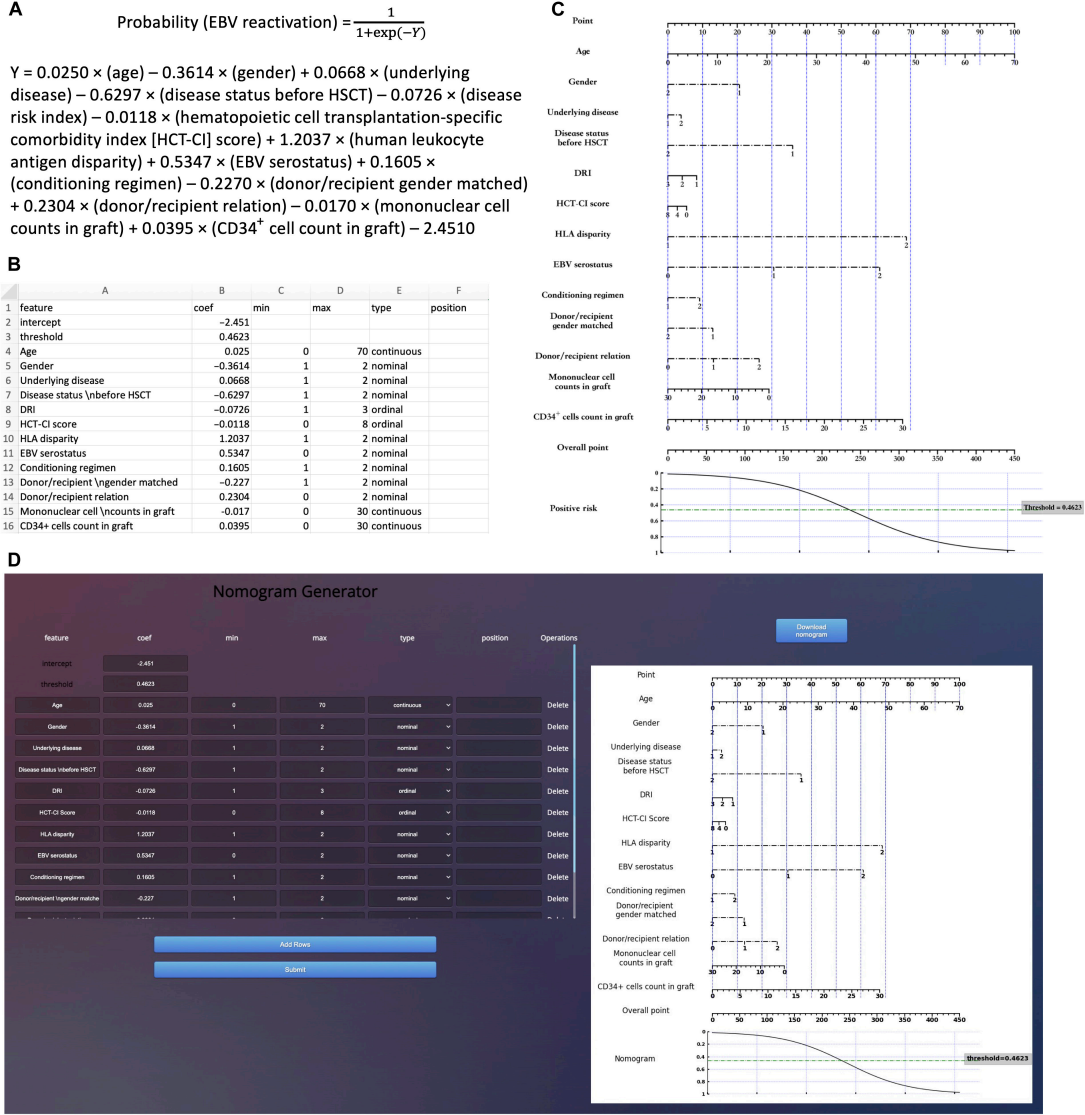
Develop nomograms from established EBV prediction model [18]. (A) The original presentation of logistic regression model information [18]. (B) The excel containing the logistic regression model information for generating nomogram. (C) The nomogram generated by simpleNomo. (D) Generate the nomogram with online generator.

**B.3 Nomogram for severe CMV prediction**

**Fig. 13.**
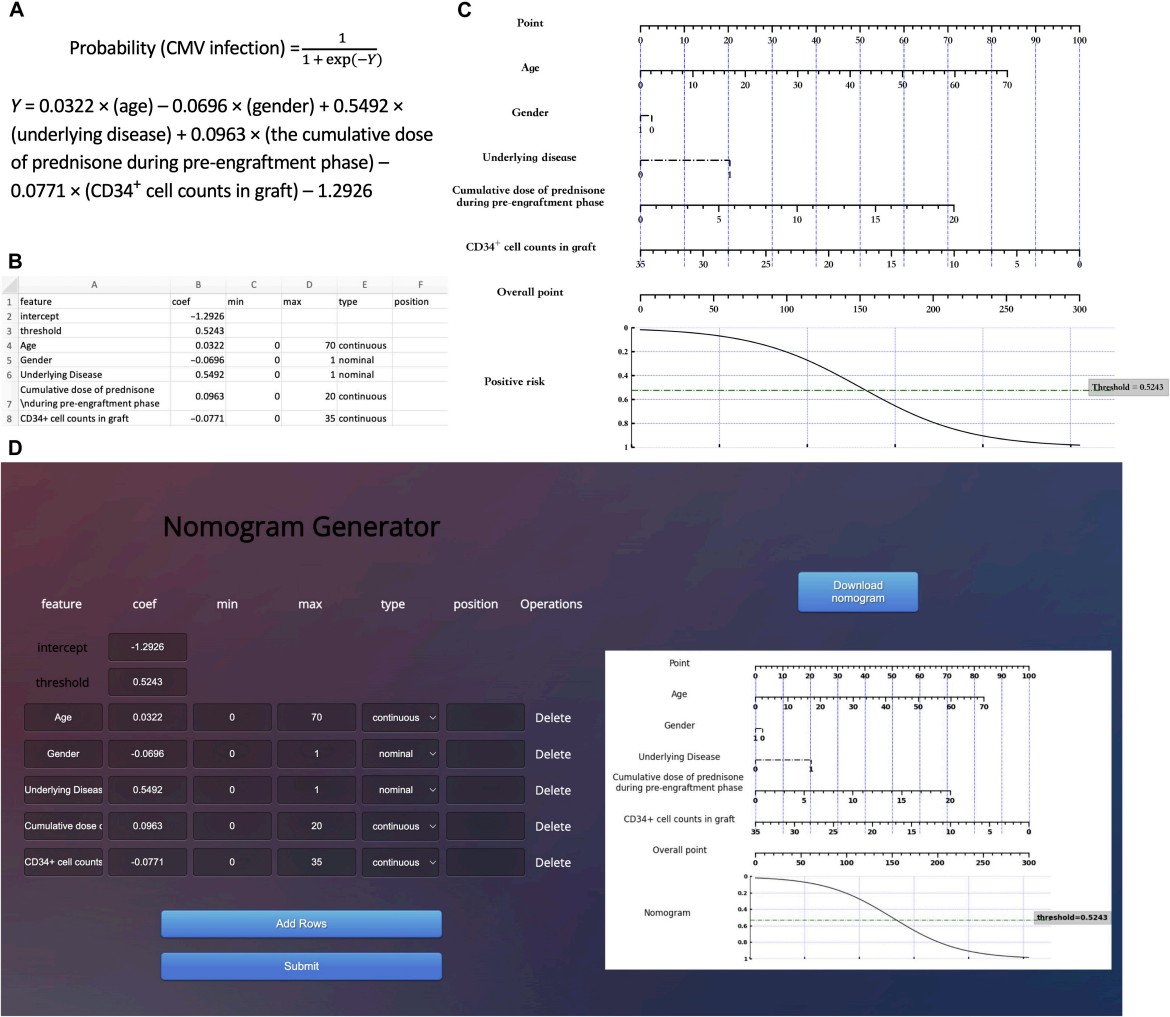
Develop nomograms from established CMV prediction model [16]. (A) The original presentation of logistic regression model information [16]. (B) The excel containing the logistic regression model information for generating nomogram. (C) The nomogram generated by simpleNomo. (D) Generate the nomogram with online generator.

**B.4 Nomogram for severe aGVHD prediction**

**Fig. 14.**
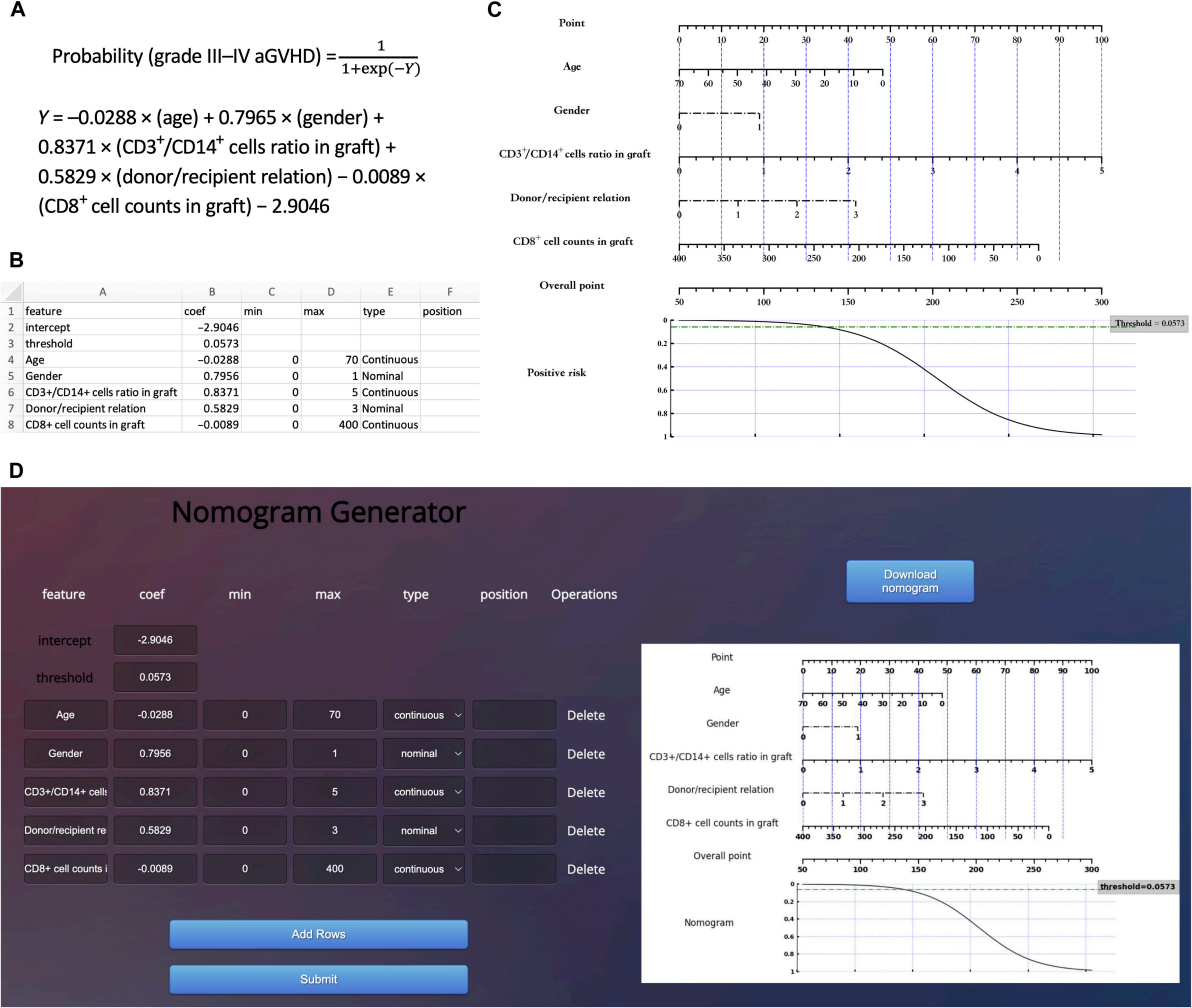
Develop nomograms from established severe aGVHD prediction model [19]. (A) The original presentation of logistic regression model information [19]. (B) The excel containing the logistic regression model information for generating nomogram. (C) The nomogram generated by simpleNomo. (D) Generate the nomogram with online generator.

**B.5 Nomogram for Risk of Mortality in Pediatric ICU prediction model**

**Fig. 15.**
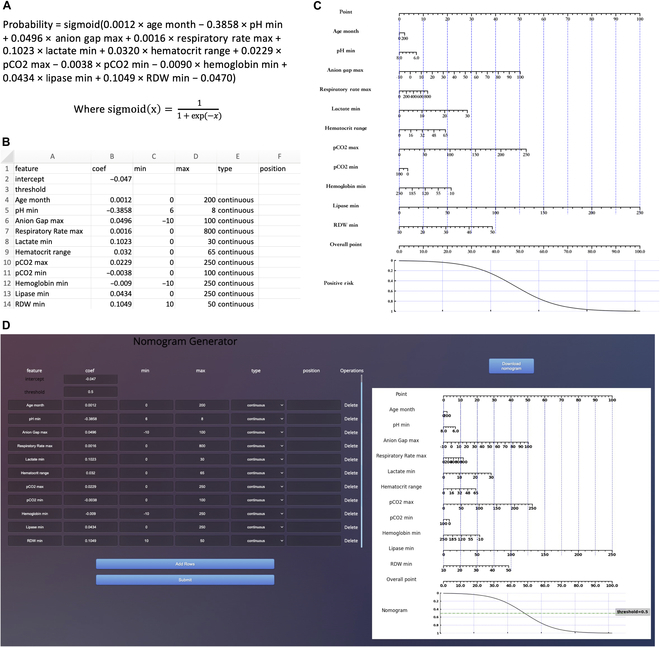
Develop nomograms from established Risk of Mortality in Pediatric ICU prediction model [20]. (A) The original presentation of logistic regression model information [20]. (B) The excel containing the logistic regression model information for generating nomogram. (C) The nomogram generated by simpleNomo. (D) Generate the nomogram with online generator.
